# Searching for New Directions for Energy Policy: Testing Three Causal Models of Risk Perception, Attitude, and Behavior in Nuclear Energy Context

**DOI:** 10.3390/ijerph17207403

**Published:** 2020-10-12

**Authors:** Byoung Joon Kim, Seoyong Kim, Sunhee Kim

**Affiliations:** 1 Department of Public Administration, Kookmin University (KMU), Seoul 02707, Korea; kimbj@kookmin.ac.kr; 2 Department of Public Administration, Ajou University, Suwon 16499, Korea; 3 Department of Local Government Administration, Gangneung-Wonju National University, Gangneung-si 25457, Korea; shkim7675@hanmail.net

**Keywords:** nuclear energy policy, risk perception, hedonic model, acceptance of nuclear power

## Abstract

Although many risk studies investigate perceptions, attitudes, and behaviors, the causal relationships among them have not yet been verified. Thus, further investigations of these relationships are necessary. This study analyzes three causal models consisting of three components: perceptions (i.e., perceived risk in this study), attitudes (i.e., satisfaction), and behavior (i.e., support for policy). This study checks these relationships in the context of nuclear energy policy. Using a hierarchical regression model, this study tests three different models between the three components: (1) Model 1 (a high-involvement model), (2) Model 2 (a low-involvement model), and (3) Model 3 (a hedonic model). First, in the high-involvement model, behavior is affected by perceptions and attitudes. In particular, attitudes mediate the relationship between risk perceptions and satisfaction. Second, in the low-involvement model, attitudes indirectly affect perceptions through behaviors. Third, in the hedonic model, behaviors affect attitudes, and risk perceptions do not mediate that relationship. This causal model does not depend on perceptions of the benefits and drawbacks of nuclear power. Our analysis shows that Model 1 is fully significant, and Model 2 and 3 are only partially significant.

## 1. Introduction

In Japan, after the Fukushima nuclear accident occurred in March 2011, the public’s acceptance of nuclear power energy decreased, and perceptions of trust in nuclear policy have become more negative [1]. However, gaps in the level of perceived risk still remain according people’s perceptions, attitudes, and behaviors [2]. One goal of current conflict management is ensuring that rational models are incorporated into the public sector in practice. However, citizens’ assessments of risky issues, such as nuclear power, are not aligned with rational resolutions. Thus, previous studies have considered many variables that may influence risk perceptions (cognition) regarding nuclear energy policy and satisfaction (attitudes) and compliance (behavior) with such policies [3,4,5]. Since many conflicts remain, better solutions to manage them are needed. Korea and Japan have faced similar problems with nuclear energy policy. Thus, a comparative study of nuclear safety can shed light on an integrated model to improve the understanding of risk communication and behavior. The central question in this study is how to acquire mediation tools for solving social conflicts. Such tools can enable citizens to use their collective intelligence, learn from each other, and prepare them to identify their own perspectives on the riskiness of nuclear power.

This study introduces two exploratory investigations. First, many behavioral economists have insisted on using bounded rational or irrational approaches to understand citizens’ actual perceptions of, satisfaction with, and support for risky alternatives. Thus, in the first exploratory study, we investigate whether people follow rational or irrational approaches. To do so, we try to determine the types of decision-making models that are involved in nuclear energy policy. We test three different patterns, high-involvement, low-involvement, and hedonic models, by using the hierarchical regression model (HRM). Currently, Korea is the fifth largest producer of nuclear energy worldwide; its 24 reactors generate about a third of its total electricity. Thus, understanding which decision-making models are used among citizens can help policymakers find better solutions for resolving nuclear energy conflicts.

Second, based on the three models mentioned above, this study aims to determine any differences between supporters of the use of nuclear power as a major energy source and the opponents. During Korea’s last presidential campaign in early 2017, President Moon pledged to phase out coal and nuclear energy, mainly owing to the public’s growing concerns about nuclear safety. Instead, he vowed to increase the share of renewable energy to 20% of total electricity generation by 2030. Because there are two different perspectives on the acceptance of nuclear energy policy, it is important to analyze the structure of the relationships between perceptions, attitudes, and behaviors among those who support and those who oppose this policy.

In this study, we first examine a series of previous studies about perceptions (i.e., perceived risk), attitudes (i.e., satisfaction with nuclear energy policy), and behaviors (i.e., the level of support for this policy). Next, we provide an overview of the research methods that are necessary to apply the HRM. After analyzing the three different models, we conclude by noting the implications of understanding the influences of these models.

## 2. Theoretical Background

### 2.1. Perceptions, Attitudes, and Behaviors

Many studies have focused on the linear relationship between attitudes and behaviors. This linear model assumes that when people perceive an object, they evaluate its properties in terms of their own beliefs. This evaluation entails a positive or negative judgment of the object and is referred to in the literature as an “attitude.” Many models representing the causal links between attitudes and actions have been suggested and proven in social science, including the theory of planned behavior (TPB), the technology acceptance model, and the norm activation model.

Fishbein and Ajzen [6] suggest the theory of reasoned action, by which greater intentions to act and actions themselves can be predicted by attitudes, such as evaluations and subjective norms. If people evaluate a specific behavior positively (attitudes) or cannot help performing the behavior (subjective norms), intentions to act are higher and actions occur more frequently. Many studies have confirmed the high correlation between attitudes and subjective norms and behavioral intentions and behaviors [7].

However, a problem with this theory is that not all intentions to act are realized by actions. Thus, it is necessary to include a variable that can explain the link between intentions to act and actions. The complementary theory is the planned theory of action [8]. According to the TPB, three factors affect behavior: attitude toward the behavior, subjective norms, and perceived behavioral control. The attitude toward the behavior is the degree to which a person evaluates or appraises the behavior in question favorably or unfavorably. Subjective norms refer to the perceived social pressure to perform or not perform the behavior. Perceived behavioral control is the perceived ease or difficulty of performing the behavior. It is assumed to reflect past experience as well as anticipated impediments and obstacles [8] (p. 188). Perceived control is the most powerful influencer of behaviors and intentions and is similar to the concept of self-efficacy, proposed by Bandura [9]. Perceived self-efficacy is a person’s evaluation of his or her ability to execute the necessary courses of action to deal with prospective situations [9]. Perceived control leads to action for two reasons. First, a greater sense of control over a specific action leads individuals to put greater effort into that action. Second, perceived control acts as a substitute for actual behavior [8].

Using hierarchical regression analyses, Ajzen and Madden [10] tested the TPB, which stresses perceived control over behavioral achievement as a determinant of intentions and behavior. The perceived sense of behavioral control refers to the degree to which individuals believe that they can control a given behavior. Ajzen and Madden [10] showed that the TPB predicted intentions and goal attainment more accurately than the theory of reasoned action did. In particular, the perception of behavioral control significantly predicted intentions [10].

One problem with the TPB is that it lacks a detailed explanation of the relationship between the variables that affect behaviors and intentions to act. In particular, it provides little explanation of the relationships among attitudes toward specific behaviors, subjective norms, and perceived control. Thus, it is necessary to examine the causal relationships between these three factors. Recently, the literature has actively discussed models that establish linear causal relationships between perceptions or beliefs, attitudes, and behavior. For example, Fischer [11] argued that linear models that assume a causal link from perceptions to attitudes to intentions and decisions and, finally, to behavior have long dominated in consumer behavior research. These linear models assume that when people perceive an object, they evaluate its properties in terms of their own beliefs. Ramond [12] suggested three variations of the basic hierarchical models: learn-feel-do, learn-do-feel, and do-feel-learn. In these models, “learn” refers to the cognitive element, which describes how and to what extent people become aware of the object’s attributes. “Feel” factor is a type of affect that describes whether people like or dislike the object. “Do” refers to the behavior toward the object.

However, this linear model has been criticized by several researchers. For example, Reibstein et al. [13] investigated the causality between perceptions, attitude, and behavior and showed that attitudes and behavior mutually influence each other. Moreover, attribute perception has a mediating effect on behavior.

These studies show that the causal relationships among perceptions, attitudes, and behavior continue to be debated. This study empirically examines the relationships among these factors in the context of nuclear energy policy.

### 2.2. The Psychometric Paradigm in Risk Studies

The dominant paradigm in previous risk studies is the psychometric one, which emphasizes perception. The psychometric paradigm was developed by Fischhof et al. [14]. It is a research approach that is used to explain how laypeople (i.e., non-experts) perceive various hazards. This paradigm asks respondents to evaluate a list of hazards using ratings scales. This process suggests that laypeople use qualitative information, such as perceptions of dreadfulness and newness, rather than more objective information, such as probabilities and other statistical information, to make intuitive risk assessments [15]. In addition, this paradigm stresses the scale that is used to measure the characteristics of the risks that are important in shaping human (risk) perceptions [16]. It treats risk perception as multidimensional and uses multidimensional scaling, clustering, and factor analysis to identify its underlying psychological dimensions [17].

The advantage of this approach is that it enables comparisons of the perceptions of different hazards. The main output of the psychometric paradigm is a cognitive map. This map is a visual representation of people’s perceptions of risk. In particular, the psychometric paradigm has shown that laypeople and experts perceive risk differently [18]. Perceptions contain various dimensions rather than just one single dimension. Darker [19] explained that perceived risk has three dimensions: perceived likelihood (i.e., the probability that an individual will be harmed by a hazard), perceived susceptibility (i.e., the individual’s constitutional vulnerability to that hazard), and perceived severity (i.e., the extent of harm that hazard would cause).

Because the psychometric paradigm approach focuses on individual perceptions, various limitations have been pointed out. First, although perception must be considered an artifact at the individual level, many studies use the value of risk perception, which is calculated at the aggregate level. However, this calculation leads to an ecological error which occurs when a value at a higher level is applied to a value at a lower level. Siegrist et al. [18] pointed out that perception is an individual-level phenomenon, requiring analyses of individual differences, but existing empirical studies are mainly conducted at the aggregate level. Marris et al. [20] showed that some of the strong intercorrelations between risk characteristics that are observed at the aggregate level are not supported when the same data are analyzed at the individual level. Similarly, Siegrist et al. [18] demonstrated that individual differences are responsible for much of the variance in that data. Specifically, over 60% of this variance is associated with individual differences or measurement errors. Thus, they suggested that laypeople’s risk perceptions cannot be explained by a model based on aggregate data.

Second, because the psychometric paradigm focuses on perceptual elements, it tends to ignore other factors, such as emotional, cognitive, and behavioral factors. Perception is the process or result of becoming aware of objects, relationships, and events using our senses. It includes such activities as recognizing, observing, and discriminating [21]. Risk perceptions in particular are beliefs about potential harm or the possibility of a loss. Because they are people’s subjective judgments about the characteristics and severity of a risk [19], they are consequently linked to cognitive processes. Emotional and cognitive factors, therefore, influence risk assessment. For example, Oh et al. [22] combined the psychometric paradigm with the impersonal- and differential-impact hypotheses to explain risk perceptions regarding H1N1. They showed that the emotional dimension of risk characteristics was positively related to risk perceptions, but the cognitive dimension was not. Moreover, media affected individual-level risk perceptions through their indirect impacts on the emotional dimension of risk characteristics.

Third, previous studies have not questioned the causal model associated with risk perceptions. Breakwell et al. [23] pointed out that although the psychometric paradigm provides an instrument for exploring individuals’ perceptions of hazards, it has limitations. It cannot necessarily explain why an individual evaluated a hazard in a particular way or why one individual perceives a hazard differently from another. This question is a matter for a causal model, which consists of cause and effect. Although many empirical studies of risk have been conducted, few causal models have been developed based on the psychometric paradigm. According to Wåhlberg [24], if a theory is defined as a statement of a causal mechanism that produces a verifiable hypothesis, then studies based on psychometric paradigm do not offer as many theories for causal research as can be expected from face value.

Our study notes the second and third limitations of the psychometric paradigm. It focuses on the perceptions, attitudes, and behaviors that have been overlooked in previous research and presents three causal models relating these three factors to existing perceptions.

### 2.3. Perceptions, Attitudes, and Behavior in Nuclear Power

Studies related to risk perceptions, attitudes, and behaviors have been conducted in the context of nuclear energy.

First, in terms of perceptions, a number of studies have investigated the impact of perceived risk on the acceptance of nuclear energy. The public perceived that nuclear energy is risky, and perceived risk is regarded as a crucial independent variable impacting the level of acceptance and policy satisfaction [25]. According to Kanda et al.’s [26] study of the perceived risk of nuclear power and other risks, Japanese people’s risk perceptions remained uniform from 1983 to 2007 irrespective of their gender, age, and occupation. Female clerical staff consistently evaluated nuclear power as the riskiest energy source during those 25 years, whereas researchers’ judgments fluctuated with global events, such as the Chernobyl accident. Citizens tended to learn how to acclimate themselves to scientific technologies with low risk levels in exchange for high benefits, except in the case of nuclear power. Because nuclear power raises the level of risk, actions against it provide benefits. Thus, nuclear power was regarded as a high-risk energy source by Japanese citizens even before the Fukushima nuclear power plant accident in March 2011.

Perceptions of radioactivity and nuclear energy facilities differ between experts and ordinary citizens. For experts, these facilities are not complex and can be easy to manage, the consequences and chances of accidents are small and manageable, and safe and technically feasible solutions exist. However, citizens are afraid of nuclear energy and perceive the facilities as dangerous, meaning that opposition to radiation facilities prevails (cf., nimbyism).

Second, nuclear power-related attitudes are conceptualized as positive or negative evaluation orientation or satisfaction. These evaluations are positive or negative judgments about the object in question, and they are referred to as attitudes [11]. Kitada [27] analyzed surveys conducted in Japan over the past 30 years and reported that the frequency of negative opinions on nuclear power generation, which ranged from 20–30% over the past 30 years, and increased to 70% four to six months after the Fukushima accident. Additionally, many people opposed future replacements or the new construction of nuclear power plants. Furthermore, when considering power generation options, people now tend to focus more on accident risk. Attitudes can be defined as the level of satisfaction with a specific object. Previous studies have focused on determining the effect of perceived risk on satisfaction with or acceptance of nuclear power [2,25].

Third, behavior is operationalized into final actions and decisions as a result of perceptions and attitudes. In nuclear power research, the support or acceptance of nuclear power is the final byproduct of perceptions and attitudes. Public acceptance of nuclear power is important for the government, the major stakeholder in the industry, because consensus is required to drive actions. It is no coincidence that the governments of nations that operate nuclear reactors endeavor to enhance public acceptance of nuclear power. Greater acceptance enables stable power generation and the peaceful processing of nuclear waste produced from nuclear reactors [28]. According to Visschers and Siegrist [29], acceptance of nuclear power before the Fukushima accident was highly correlated with acceptance after the accident. Thus, the accident had only a moderate impact on acceptance. In Korea, a keyword analysis of nuclear power showed that the public had positive attitudes toward nuclear power when the country successfully exported nuclear reactors to the United Arab Emirates (UAE). With the Fukushima accident in 2011 and supplier scandals in 2012, however, nuclear power’s image was degraded, and that negative image continues [28]. As mentioned, the level of acceptance of nuclear energy is used as a major dependent variable in previous studies [1,4,29,30]. Moreover, one study of causal relationship models shows that affect (i.e., prior attitude) influences evaluations of the government, trust in regulations, and acceptance of public policy [31].

Most previous studies of different risks may be criticized for not specifying the assumed causal relations between the variables [32]. Recently, however, risk studies have taken more interest in causal models. By using structural equation modeling to examine the causal factors affecting acceptance, Wang and Li [33] showed that the perceived energy supply benefit was the most important factor. Moreover, the perceived environmental benefit significantly positively affected support, whereas the perceived risk level affected support negatively. Ryu et al. [1] found that prior attitudes are related to the perceived risk of nuclear power energy and acceptance of nuclear power.

## 3. Materials and Method

### 3.1. Research Model and Analysis Method

This study does not take a traditional rational approach. Instead, by using an FCB (Foote, Cone and Belding) grid [34] and other behavioral economists’ approaches, it aims to find more reliable and practical implications for nuclear energy policies [35]. Based on the decision-making processes used by marketing or public relations studies, this study considers high-involvement (thinking), low-involvement (thinking), and hedonic models.

**Figure 1:**
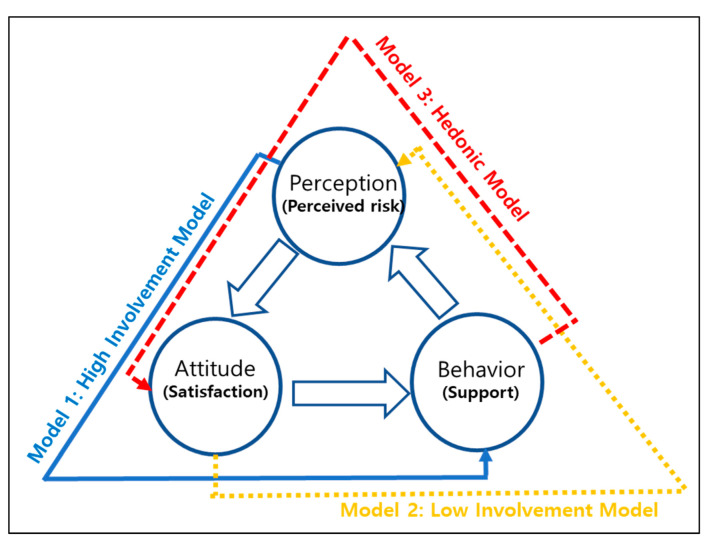
Conceptual framework.

Figure 1 summarizes the research framework for this study, which is based on three models (Model 1: high-involvement model, Model 2: low-involvement model, and Model 3: hedonic model). Essentially, it includes perceptions (i.e., perceived risk), attitudes (i.e., satisfaction), and behavior (i.e., support for the policy). The control variables include gender, age, education level, and income.

Model 1 (the high-involvement model) assumes that relationship between perceptions, attitudes, and behavior is linear. In this model, decisions are made carefully, meaning that the thinking process is sequential. Highly involved decision-making is linked to an individual’s inner ego, self-image, and risk. It is natural for the general public to consider various risks in the policy evaluation process. In contrast, in the low-involvement model, a final decision is made based on an immediate judgment. Behavior, the result of this judgment, provides the basis for perception. Thus, Model 2 (the low-involvement model) is repetitive, low-risk, and habitual. In this case, risk factors are not considered in decision-making.

Model 3 (the hedonic model) assumes that behavior occurs before an accident, based on habitual behavior. A seminal work by Festinger and Carlsmith [36] showed that experience of cognitive dissonance can change an individual’s attitudes. They conducted an important experiment that demonstrated the extent to which behaviors that contradicted an individual’s initial beliefs could create cognitive dissonance and influence attitudes. Sometimes, behavior is the criterion that is used to judge attitudes. According to Bem’s [37] self-perception theory, people use their behavior to predict their attitudes when they do not know their attitudes very well. People determine their attitudes by observing their own behavior and deciding what attitudes must have led to this behavior.

Because these three models have never been verified at the same time, this study attempts to do so and determine their implications. The main contents of the three models are shown in Table 1.

The high-involvement model has been verified in a previous study. By integrating the risk perception attitude framework and the TPB, Shi and Kim [38] showed that perceived risk interacted with self-efficacy to affect behavioral intentions to seek counseling. However, this interaction was only observed among individuals with favorable attitudes toward counseling-seeking behavior and was not found among those with unfavorable attitudes. However, neither the low-involvement model nor the hedonic model has been verified.

The analytical methods used in this study include mean and regression analysis. In the mean analysis, we analyze how the average values of perceived risk of, satisfaction with, and support for nuclear power differ across demographics, such as education level, gender, age, and household income. In the regression analysis, the relationships between perceptions, attitudes, and behaviors are analyzed, focusing on the three variables’ mediating roles. We use hierarchical regression analysis to estimate the mediating effects.

### 3.2. Measure and Reliability Analysis

We define three dependent variables for the study. Perception–Perceived Risk reflects citizens’ reported perceptions of the riskiness of nuclear energy. Attitude–Satisfaction with Nuclear Policy reflects citizens’ reported attitudes toward nuclear energy. Behavior–Support for Nuclear Energy reflects citizens’ reported actions. The three dependent variables are constructed as follows.

Perception–Perceived Risk: this variable is measured based on the answers to the following four questions: How much do you agree with (1) the level of risk of using nuclear plants to provide electricity, (2) the level of risk of regional conflicts, (3) the level of accidents in nuclear facilities, and (4) the overall level of danger of nuclear energy (scale: 1 = not agree at all, 2 = not agree, 3 = neutral, 4 = agree, 5 = strongly agree). The answers to these questions are combined into an additive construct (Cronbach’s α = 0.733).

Attitude–Satisfaction with Nuclear Policy: this notion is measured by the answers to the following five questions; Currently, how do you rate: (1) the level of affection toward the overall system and policies, (2) the current level of satisfaction with nuclear policy in terms of safety, (3) the current level of satisfaction with nuclear policy in terms of usability, (4) the current level of satisfaction with nuclear policy in terms of economics, and (5) the current level of satisfaction with nuclear policy in terms of environmental friendliness (scale: 1 = not satisfied at all, 2 = not satisfied, 3 = neutral, 4 = satisfied, 5 = strongly satisfied). The answers to these questions are combined into an additive construct (Cronbach’s α = 0.832).

Behavior–Support for Nuclear Energy: this notion is measured by the answers to the following five questions: (1) how much are nuclear facilities needed (scale: 1 = not needed at all, 2 = not needed, 3 = neutral, 4 = needed, 5 = strongly needed); (2) how much do you agree that nuclear facilities are important tools for generating electricity (scale: 1 = not agree at all, 2 = not agree, 3 = neutral, 4 = agree, 5 = strongly agree); (3) what is the desirable level of nuclear plants (scale: 1 = must reduce, 2 = somewhat reduce, 3 = neutral, 4 = somewhat increase, 5 = must increase); (4) how much do you agree with constructing nuclear facilities near your community (scale: 1= not agree at all, 2 = not agree, 3 = neutral, 4 = agree, 5 = strongly agree); and (5) overall, I support nuclear energy as the most usable energy resource (Scale: 1 = not agree at all, 2 = not agree, 3 = neutral, 4 = agree, 5 = strongly agree). The answers to these questions are combined into an additive construct (Cronbach’s α = 0.922).

Table 2 provides further details about the measures. All of the variables are represented by variables that were subjected to reliability analysis. The survey questions were selected such that the constructs are as similar as possible to those reported in prior studies [2,5,25].

### 3.3. Data Collection

This study is based on data taken from a national online survey of citizens living in South Korea. The data were collected in June 2017. The online survey (structured questionnaire) was called “Nuclear Power Policy Survey for the Public.” It measured the perceived risk of nuclear energy, attitudes on nuclear energy policy, the level of acceptance on nuclear power, and other issues related to nuclear energy policies. The survey targeted adult men and women aged 19 to 59 years. A total of 700 people were surveyed through an online panel survey method. Table 3 shows the distribution of respondents by gender, age, income, and education as well as the average values of perceived risk of, satisfaction with, and support for nuclear energy.

### 3.4. Research Procedure

In the first stage of the research procedure, we reviewed theories on the relationships among perceptions, attitudes, and behaviors. In the second stage, a questionnaire survey was conducted to collect the necessary data for this study. The survey items leveraged measurement questions used by previous studies. To ensure the reliability and validity of the questions, we conducted experts’ cognitive test and public’s response test. In the third stage, we conducted the analysis, focusing on the mediating relationships among perceptions, attitudes, and behaviors. In the fourth stage, we discuss the relevance of and issues with existing research results.

## 4. Results

### 4.1. Three Models

This study employs the HRM to test the relative influences of individual-level factors (i.e., gender, age, education, and income) on three components. In the first step, the demographic control variables and the independent variables are entered in the first block, and the mediating variable is entered in the dependent variable (Regression 1). In the second step, the demographic control variables and the independent variables are entered in the first block (Regression 2). Finally, the demographic and independent variables are entered in the first block, and the mediation variable is entered in the second block (Regression 3). In particular, this study aims to detect the effect of mediation in each model. The results are shown for different values of the dependent variable in Table 4, Table 5 and Table 6. Table 4 shows the results of testing the high-involvement model.

In Table 4, all relations are statistically significant, and perception is negatively related to attitudes and behaviors. In addition, attitudes mediate the relation between perceptions and behaviors. The finding that perceptions are negatively related to attitudes and behaviors indicates that a higher level of perceived risk reduces positive attitude toward nuclear energy and the level of support for nuclear power. The mediating variable in this model, attitudes, is positively related to behavior. Thus, an individual who has a more positive attitude toward nuclear power is more likely to accept nuclear power as a major energy source for generating electricity. Thus, positive attitudes toward nuclear policy positively mediate the relationship between the level of perceived risk and the level of support. This finding is consistent with findings from previous studies. The fact that support for nuclear facilities is created by the mediation of perceptions (perceived risk) and attitudes (policy satisfaction) implies that perceptual and attitudinal factors must be considered simultaneously when establishing nuclear policies. In addition, to induce support for facilities, policymakers must recognize that perceptions (perceived risk) affect not only behaviors (nuclear support) but also attitudes (policy satisfaction).

Table 5 presents the results for the low-involvement model.

Table 5 shows that attitudes indirectly affect perceptions via behavior (i.e., the level of support). A greater value for attitude implies a more positive attitude toward nuclear power. A greater value of behavior implies greater acceptance of nuclear energy for generating electricity. Thus, the negative indirect effect makes sense in this model. In addition, the low-involvement model partially explains citizens’ decision-making process. Thus, citizens mainly follow the rational approach but also partially use the irrational approach.

This analysis shows that perceptions (perceived risk) are affected by attitudes (policy satisfaction) but only through behaviors (acceptance of nuclear power). The full mediating effect of behavior suggests that various efforts and strategies considering behavioral factors are required to enhance acceptance of nuclear power.

Next, we check whether the hedonic model can explain citizens’ decision-making process regarding nuclear power. Table 6 presents the results for the hedonic model.

From Table 6, we find no mediating effect of perceptions on attitudes. Thus, behavior and attitudes are positively connected. If an individual takes a positive action toward nuclear energy, such as accepting the nuclear facilities near his or her community, the individual’s level of support increases. These results imply that behavioral experiences within a community can increase support for nuclear power regardless of perceived risk.

In the hedonic model, behavior (nuclear support) directly influences attitude (policy satisfaction). However, perception (perceived risk) does not mediate the relationship between behaviors and attitudes. Thus, to increase policy satisfaction, it is necessary to draw out support for nuclear power.

The results of this analysis show that there is no mediating effect of attitude in the high-involvement model. We find that behavior has a full mediating effect in the low-involvement model and in the hedonic model. These three different results suggest the possibility of various causal models.

### 4.2. Three Models of Supporting and Opposition Groups

Korea’s current government under President Moon has changed the direction of its major nuclear policy from expanding to reducing the proportion of nuclear power used in generating electricity. We split the sample into these two groups (i.e., anti-group and pro-group) to examine how the structures of the three models differ across them. We define the “anti-group” (N = 485) as respondents who support a policy of denuclearization, and we define the “pro-group” (N = 215) as respondents who support maintaining current nuclear policy.

**Figure 2:**
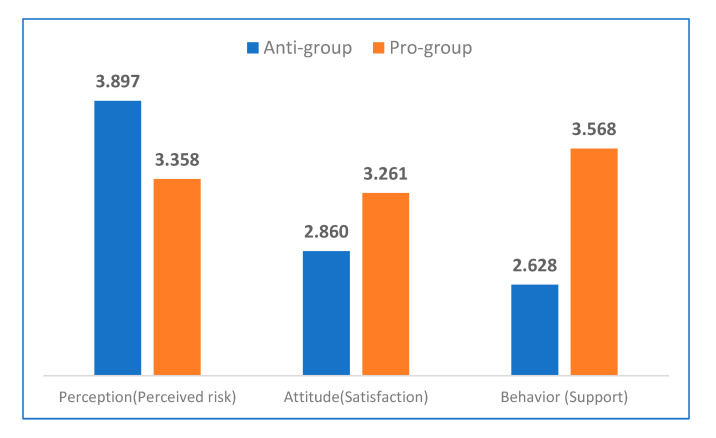
Means of perceived risk, satisfaction, and support in the pro-group and the anti-group.

Figure 2 shows the differences in perceived risk, satisfaction, and behavior across the anti-group and the pro-group. First, the group that favors nuclear power has lower risk perception levels than the group that opposes it (F-value = 116.751, *p*-value = 0.000). In terms of satisfaction and support, the group that favors nuclear energy has a higher score than the opposition group has, and this difference is statistically significant (F-value = 58.386, *p*-value = 0.000; F-value = 227.457, *p*-value = 0.000).

The results of the three models differ for these two groups with different perspectives on future energy policy.

Table 7 showed that citizens who view the nuclear policy of Moon’s current government positively use the high-involvement model as their main decision-making model. In the anti-group, perception has a negative effect on the level of support (behavior), and this effect is statistically significant. Thus, the level of support behavior is influenced by politics. The directions in the model may change depending on the country’s political circumstances. Interestingly, even among people who oppose nuclear power, higher satisfaction levels imply greater support for nuclear power. Thus, nuclear power policy offers a positive utility to the public, and people can form a positive attitudes toward nuclear power.

Next, we explore differences in the low-involvement models for the two groups. Table 8 presents the results. Among citizens who support the current government policy (reducing nuclear power), attitudes affect perceptions via behavior (indirect effect). Thus, the low-involvement model also describes citizens’ decision-making processes regarding nuclear energy. Even citizens who do not support the current policy (reducing nuclear power) show a similar pattern. These findings show that low involvement is one of factors in the dynamics of citizens’ reasoning. Thus, policymakers should consider this approach to understand and provide better solutions to this type of conflict.

Finally, Table 9 shows the comparison of the anti-group and the pro-group in terms of the hedonic model. Again, both groups have similar patterns.

Table 10 highlights and summarizes the findings of this study.

## 5. Conclusions

This study highlights the usefulness of considering irrational approaches to describe citizens’ decision-making processes regarding nuclear energy policy. Prior studies have only examined the connections among perceptions, attitudes, and behavior using a rational approach and have not used different investigation methods to explore their relationships. Citizens may make decisions regarding nuclear energy policy based on thinking and rational reasoning by, for example, digesting facts and then choosing to have a positive or negative attitude. Based on their rational judgments, they choose their level of acceptance, that is, their behavior.

However, individuals may take action first and then think about their perception of risk. They may develop their attitudes toward nuclear energy without thinking of risk or may have other preexisting attitudes toward nuclear power. Individuals may take actions and then determine their attitudes. These different ways of thinking have not been considered important in previous studies of nuclear energy policy. In addition, few studies have investigated this issue other than Jeon et al. [25]. They argued that individual citizens who set their attitudes and beliefs before seeking correct information differ demographically from the rest of the population. Individuals with large psychological deviations from the population at large, such as more risk-averse or risk-taking behaviors, exhibit different behaviors [25]. For example, even if all of the sub-items for policy satisfaction were high, meaning that citizens said that they were very satisfied, the majority of citizens utilized risk-averse strategies to make judgments. The weakest level of satisfaction in a certain area could be the critical factor in taking positive action toward nuclear energy issues.

This study found several key results. First, in the high-involvement model, people’s behavior (i.e., nuclear power support) is affected by perceptions (i.e., perceived risk) and attitudes (i.e., satisfaction). In particular, attitudes mediate the relationship between risk perception and satisfaction. Second, in the low-involvement model, attitudes (i.e., policy satisfaction) do not directly affect perceptions (i.e., perceived risk). Instead, they indirectly affect risk perception through behavior (i.e., support for nuclear energy). Third, in the hedonic model, behavior (i.e., support for nuclear energy) affects attitudes (satisfaction), but perceptions (risk perception) do not mediate this relationship.

The three models can be used as general explanatory models in that their results are consistent among people that favor nuclear power and people that oppose nuclear power. The verification results for the three models show that attitudes have mediating effect in the high-involvement model. However, they find full mediation by behavior in the low-involvement model and in the hedonic model. These three different results suggest the possibility of various possible causal models explaining the relationships among perceptions, attitudes, and behavior.

## 6. Discussion and Limitation

### 6.1. Discussion

This study attempted to verify the causal relationships between attitudes and behaviors and between perceptions and attitudes as well as the reverse causal relationships among those variables. In general, attitudes are thought to affect behaviors, but recent studies have noted that behaviors influence attitudes as well. Sussman and Gifford [39] suggested that in the TPB, reverse causal influences of intentions on base components (i.e., attitudes toward behaviors, subjective norms, and perceived behavioral control) may exist. Empirical studies find that attitudes and behaviors interact or that behaviors change attitudes. Regarding these interactive relationships, Reibstein et al. [14] investigated the causal relationships among perceptions, affect, and behavior. They showed that attitudes and behaviors mutually influence each other. The frequency of using the bus positively affects attitudes toward using the bus. Moreover, based on hypotheses on reverse causality, van Wee et al. [40] assumed that behavioral influences (e.g., travel behavior) could change attitudes. Golob [41] also showed that behaviors influenced attitudes. For example, carpool use negatively shapes both attitudes toward fairness to carpoolers and perceptions of effectiveness, whereas FasTrak positively shapes attitudes toward approval. Ziegler and Schlett [42] empirically validated that past extra-role behavior is significantly related to job satisfaction given low work centrality. Kroesen et al. [43] assumed that behavior influences attitudes more than attitudes influence behavior; for example, dissonant travelers are more likely to change their attitudes than their behavior. This study confirms that attitudes do affect behavior. Based on the results, the question of why behavior can change attitudes may arise. Experiences are the direct factors that influence attitude changes. One theory that explains how attitudes change behavior is cognitive dissonance theory. This theory explains that dissonance between attitudes and behavior causes one of them to change [44].

This study also analyzed the effect of attitudes on perception. Although it is unreasonable to argue that perceptions change behavior, empirical evidence shows that the two are related. For example, Fazio and Williams [45] showed that individuals’ attitudes guide their subsequent perceptions of objects, which is a function of the accessibility of those attitudes from memory. Moreover, Tyagi and Wotruba [46] found reverse causality between perceptions and behavior; behavior influences perceptions. The behavioral intention to quit one’s job is more likely to impact the perceptions of certain variables, such as the organizational climate, job satisfaction, and organizational commitment. According to Harter et al. [47], managerial actions and practices can impact employees’ work conditions and their perceptions of these conditions. However, this study does not confirm a direct influence of attitudes on perceptions. We did find that attitudes indirectly affect perceptions through behavior. In short, we showed that the relationships between attitudes and behavior, perceptions and behavior, and perceptions and attitudes can be identified not only as causal relationships but also as reverse causal relationships.

When we compare the high- and low-involvement models, the high-involvement model is functional. When people judge the issue of nuclear energy, they rely on existing perceptions to make reasonable judgments, and these perceptions lead to satisfaction and support. The higher the perceived risk is, the lower the satisfaction with nuclear policy is. Support for nuclear power declines in turn as well. In terms of the results, risk perception plays an important role in nuclear satisfaction and support. Because perceived risk is inversely related to perceived benefits, an appropriate combination of safety and benefit policies can affect public attitudes toward nuclear power. Regarding the hedonic model, our findings show that behavior can influence attitudes. The impact of behavior on attitudes suggests the importance of people’s everyday experiences. The Fukushima nuclear accident was a negative experience and decisively negatively affected people’s attitudes toward nuclear power. Similar negative similar events in the future will also change people’s attitudes.

Finally, the analysis shows that the causal structure is basically the same for people who support and oppose nuclear power. Interpretation is difficult because the two groups show the same determinant structure. Interestingly, even in the group opposing nuclear power, higher satisfaction leads to greater support for nuclear power. This result suggests that nuclear policy gives individuals some positive utility, which can lead to positive attitudes toward nuclear power.

### 6.2. Limitation

One limitation of this study is that the data are snapshots of a certain period of time. Second, although the study assumes that the measures of the three components causally impact each another, the directions of the causality may be reversed [48]. Thus, the models may be matched in different ways. Overall, this study’s findings help to expand the understanding of citizens’ decision-making processes regarding nuclear policy. As this study progressed, more research questions of interest were generated. To investigate the latent relationships among the three major component variables more deeply, we will attempt to use the structure equation model in future research. Additionally, other factors, such as trust in government, political preferences, and social solidarity, may impact the relationships in the investigated models in this study. Investigating these factors may also be an interesting topic for future study.

Moreover, because we only focused on perceptions, attitudes, and behavior, value factors were dismissed. Because various values and cultures exist in the world [49,50,51,52,53,54,55,56], the role of values needs to be examined in terms of the relationships among perceptions, attitudes, and behavior. Lastly, structural context, resource, and communication factors may matter in the relationships between the three variables [56,57,58,59,60,61,62,63], but we did not consider them when we set up the models. Overall, future research can contribute to the understanding of citizens’ risk communication and behavior.

## Figures and Tables

**Table 1:** Summary of three models.

	Model 1: High-Involvement Model	Model 2: Low-Involvement Model	Model 3: Hedonic Model
Causality	Perception → Attitude → Behavior	Attitude → Behavior → Perception	Behavior → Perception → Attitude
Dependent variable	Behavior	Perception	Attitude
Thinking process	Sequential	Sequential/Reverse	Reverse
Locus control	Internal	Internal	More external
Mode of Decision making	Considerate, high-risk, and new ways	Repetitive, low-risk, and habitual	Post-hoc thinking

**Table 2:** Measures and reliability test.

Composite Name	Alpha Values	Survey Questions
Perception–Perceived Risk	0.733	How much do you agree with: (1) the level of risk of using nuclear plants to provide electricity. (2) the level of risk of regional conflicts. (3) the level of accidents in nuclear facilities. (4) the overall level of danger of nuclear energy.
Attitude–Satisfaction with Nuclear Policy	0.832	Currently, how do you rate: (1) the level of affection toward the overall system and policies. (2) the current level of satisfaction with nuclear policy in terms of safety. (3) the current level of satisfaction with nuclear policy in terms of usability. (4) the current level of satisfaction with nuclear policy in terms of economics. (5) the current level of satisfaction with nuclear policy in terms of environmental friendliness.
Behavior–Support for Nuclear Energy	0.922	(1) How much do you resist the construction of nuclear facilities near your community? (2) Overall, I support nuclear energy as the most usable energy resource (3) How much are nuclear facilities needed? (4) How much do you accept nuclear facilities as important tools for generating electricity? (5) What is the desirable level of nuclear plants?

**Table 3:** Distribution of respondents and the mean values of perceptions, attitudes, and behaviors.

Category		Number (%)	Mean of Perception (Perceived Risk) (S.D.)	Mean of Attitude (Satisfaction) (S.D.)	Mean of Behavior (Support) (S.D.)
Education	Less than high school	108 (15.4%)	3.60 (0.654)	3.11 (0.619)	3.10 (0.805)
	University	524 (74.9%)	3.75 (0.653)	2.95 (0.675)	2.88 (0.871)
	Graduate school	68 (9.7%)	3.76 (0.682)	3.01 (0.656)	2.88 (0.987)
Gender	Male	353 (50.40%)	3.66 (0.677)	3.07 (0.692)	3.04 (0.952)
	Female	347 (49.62%)	3.80 (0.630)	2.90 (0.629)	2.79 (0.772)
Age (years)	Less than 29	163 (23.3%)	3.73 (0.600)	3.04 (0.637)	2.91 (0.768)
	30–39	164 (23.4%)	3.77 (0.715)	2.88 (0.684)	2.79 (0.928)
	40–49	194 (27.7%)	3.68 (0.627)	2.93 (0.649)	2.86 (0.816)
	over 50	179 (25.6%)	3.75 (687)	3.09 (0.679)	3.10 (0.956)
Household income (million won)	≤200	171 (24.4%)	3.68 (0.648)	2.90 (0.628)	2.81 (0.841)
	200–399	245 (35.0%)	3.80 (0.682)	3.00 (0.680)	2.92 (0.840)
	400–699	162 (23.1%)	3.72 (0.650)	2.94 (0.647)	2.94 (0.882)
	≥700	122 (17.4%)	3.69 (0.626)	3.12 (0.703)	3.02 (0.974)
	Total	700 (100.0%)	3.73 (0.658)	2.98 (0.667)	2.92 (0.876)

Note: S.D. is standard deviation.

**Table 4:** Testing the high-involvement model.

Step	Independent Variable	Dependent Variable	Unstandardized B	Standardized Beta	t-Value	*p*-Value	R^2^
Step 1	Perception	Attitude	−0.342	−0.338	−9.527	0.000 **	0.148
Step 2	Perception	Behavior	−0.717	−0.539	−17.168	0.000 **	0.332
Step 3 (Independent Variable)	Perception	Behavior	−0.459	−0.345	−13.565	0.000 **	0.612
Step 3 (Mediating Variable)	Attitude		0.754	0.574	22.400	0.000 **	

Control variables: gender, age, education, income. ** *p* < 0.01.

**Table 5:** Testing the low-involvement model.

Step	Independent Variable	Dependent Variable	Unstandardized B	Standardized Beta	t-Value	*p*-Value	R^2^
Step 1	Attitude	Behavior	0.909	0.692	25.553	0.000 **	0.509
Step 2	Attitude	Perception	−0.338	−0.343	−9.527	0.000 **	0.135
Step 3 (Independent Variable)	Attitude	Perception	0.077	0.078	1.757	0.079	0.317
Step 3 (Mediation Variable)	Behavior		−0.457	−0.608	−13.565	0.000 **	

Control variables: gender, age, education, income. ** *p* < 0.01.

**Table 6:** Hedonic model.

Steps	Independent Variable	Dependent Variable	Unstandardized B	Standardized Beta	t-Value	*p*-Value	R^2^
Step 1	Behavior	Perception	−0.415	−0.553	−17.168	0.000 **	0.314
Step 2	Behavior	Attitude	0.553	0.700	25.553	0.000 **	0.504
Step 3 (Independent Variable)	Behavior	Attitude	0.557	0.732	22.400	0.000 **	0.506
Step 3 (Mediation Variable)	Perception		0.057	0.057	1.757	0.079	

Control variables: gender, age, education, income. ** *p* < 0.01.

**Table 7:** High-involvement models for the anti-group and pro-group for reducing nuclear energy.

Steps	Independent Variable	Dependent Variable	Standardized Beta		t-Value		*p*-Value		R^2^	
			Anti-Group	Pro-Group	Anti-Group	Pro-Group	Anti-Group	Pro-Group	Anti-Group	Pro-Group
Step 1	Perception	Attitude	−0.307	−0.109	−7.073	−1.670	0.000 **	0.096	0.124	0.155
Step 2	Perception	Behavior	−0.497	−0.241	−12.513	−4.104	0.000 **	0.000 **	0.280	0.320
Step 3 (Independent Variable)	Perception	Behavior	−0.316	−0.182	−9.868	−3.843	0.000 **	0.000 **	0.569	0.564
Step 3 (Mediation Variable)	Attitude		0.575	0.538	17.920	10.707	0.000 **	0.000 **		

Control variables: gender, age, education, income. ** *p* < 0.01.

**Table 8:** Low-involvement models for the anti-group and the pro-group.

Steps	Independent Variable	Dependent Variable	Standardized Beta		t-Value		*p*-Value		R^2^	
			Anti-Group	Pro-Group	Anti-Group	Pro-Group	Anti-Group	Pro-Group	Anti-Group	Pro-Group
Step 1	Attitude	Behavior	0.672	0.560	20.093	10.957	0.000 **	0.000 **	0.482	0.533
Step 2	Attitude	Perception	−0.308	−0.120	−7.073	−1.670	0.000 **	0.096	0.120	0.069
Step 3 (Independent Variable)	Attitude	Perception	0.052	0.083	0.963	0.947	0.336	0.345	0.269	0.131
Step 3 (Mediation Variable)	Behavior		−0.536	−0.363	−9.868	−3.842	0.000 **	0.000 **		

Control variables: gender, age, education, income. ** *p* < 0.01.

**Table 9:** Hedonic models for the anti-group and the pro-group.

Steps	Independent Variable	Dependent Variable	Standardized Beta		t-Value		*p*-Value		R^2^	
			Anti-Group	Pro-Group	Anti-Group	Pro-Group	Anti-Group	Pro-Group	Anti-Group	Pro-Group
Step 1	Behavior	Perception	−0.501	−0.309	−12.513	−4.104	0.000 **	0.000 **	0.268	0.127
Step 2	Behavior	Attitude	0.681	0.652	20.093	10.957	0.000 **	0.000 **	0.475	0.456
Step 3 (Independent Variable)	Behavior	Attitude	0.699	0.668	17.920	10.797	0.000 **	0.000 **	0.476	0.458
Step 3 (Mediation Variable)	Perception		0.037	0.052	0.963	0.947	0.336	0.345		

Control variables: gender, age, education, income. ** *p* < 0.01.

**Table 10:** Summary of findings.

Model	Hypotheses	All	Anti-Group	Pro-Group
High-Involvement Model	Independent: Perception (−) Mediating: Attitude (+)	− +	− +	− +
Low-Involvement Model	Independent: Attitude (−) Mediating: Behavior (−)	* NS −	* NS −	* NS −
Hedonic Model	Independent: Behavior (+) Mediating: Perception (−)	+ * NS	+ * NS	+ * NS

* NS = not significant.
